# ELDER MISTREATMENT AND COGNITIVE FUNCTIONING: FINDINGS AMONG CHINESE OLDER ADULTS IN THE US

**DOI:** 10.1093/geroni/igae098.0168

**Published:** 2024-12-31

**Authors:** Guoping Jin, Fengyan Tang

**Affiliations:** Adelphi University, Pittsburgh, Pennsylvania, United States; University of Pittsburgh, Wexford, Pennsylvania, United States

## Abstract

Elder mistreatment disproportionately affects racial and ethnic minority older adults, particularly Chinese older adults in the U.S., who face increased risks due to cultural disparities, intergenerational conflicts, and socioeconomic disparities. While elder mistreatment is known to compromise cognitive health as a stressful life event, research in racial/ethnic minority populations is limited. This study aimed to address this gap by investigating the longitudinal effects of elder mistreatment on cognitive decline over time among older Chinese Americans. Latent growth curve modeling (LGCM) was applied in a sample of Chinese older adults residing in the Greater Chicago area, drawn from the population Study of Chinese Elderly in Chicago (2011-2019), comprising 2,811 participants across four waves of assessment. Elder mistreatment was assessed using a comprehensive instrument at baseline and follow-ups to evaluate its prevalence and incidence, respectively. Cognitive performance tests were administered across four waves to track change trajectory. Compared with respondents without mistreatment reports at baseline, those reporting mistreatment exhibited better cognitive functioning (B = 0.07, p<.05). Those who reported being mistreated at follow-ups showed a faster cognitive decline (B = -0.04, p<.05). Neither mistreatment prevalence nor incidence was associated with an accelerated rate of cognitive decline. The results indicate that recent and potentially cumulative experiences of elder mistreatment have a negative effect on cognitive decline, underscoring the critical need for prevention, detection, and mitigation of elder mistreatment. Furthermore, culturally tailored practices and policies are needed to address elder mistreatment issues within the Chinese minority community.

